# Grazing-Incidence X-ray Scattering at High Energies: Advanced Capabilities at the 11-ID Beamlines of the APS-U

**DOI:** 10.1063/4.0001053

**Published:** 2025-10-27

**Authors:** Justin M Hoffman, Olaf J Borkiewicz, Wenqian Xu, Andrey A Yakovenko

**Affiliations:** Argonne National Laboratory, Lemont, IL, USA

## Abstract

Grazing-incidence x-ray scattering at high energies (60-130 keV) has recently been realized for structural characterization of thin films, with the 11-ID beamlines at the APS showcasing some of the first experiments. Now, the enhanced brilliance of the APS-U allows for faster collection times and greater sensitivity to fully utilize the particular capabilities of grazing-incidence x-ray scattering exclusively available at high energies. Specifically, collection of total x-ray scattering data for pair distribution function analysis allows for determination of local structure in amorphous and disorder films, including orientational resolved local structure. Additional functionalities not available at lower energies will be demonstrated including “inverse” depth profiling to characterize buried interfaces and increased accessibility of the *q* space near the vertical *qz* axis. Capabilities for *in-situ* and *operando* experiments such as variable-temperature experiments and measurements during film growth will be discussed as well. These experiments will benefit from the increased flux of the APS-U by allowing for time resolution on the order of milliseconds to study fast processes. Overall, it is expected that grazing-incidence x-ray scattering at high energies will be applicable to a wide variety of fields and sample environments.

